# Unravelling the Molecular Dialogue of Beneficial Microbe−Plant Interactions

**DOI:** 10.1111/pce.15245

**Published:** 2024-11-04

**Authors:** Ashish K. Srivastava, Reema D. Singh, Girdhar K. Pandey, Prasun K. Mukherjee, Christine H. Foyer

**Affiliations:** ^1^ Nuclear Agriculture and Biotechnology Division Bhabha Atomic Research Centre Mumbai Maharashtra India; ^2^ Homi Bhabha National Institute Mumbai India; ^3^ Department of Plant Molecular Biology University of Delhi South Campus New Delhi India; ^4^ School of Biosciences University of Birmingham, Edgbaston Birmingham UK

**Keywords:** host‐range specificity, microbial transplantation, plant−microbe communication, reactive oxygen species, receptor−ligand signalling, root microbiome

## Abstract

Plants are an intrinsic part of the soil community, which is comprised of a diverse range of organisms that interact in the rhizosphere through continuous molecular communications. The molecular dialogue within the plant microbiome involves a complex repertoire of primary and secondary metabolites that interact within different liquid matrices and biofilms. Communication functions are likely to involve membrane‐less organelles formed by liquid−liquid phase separation of proteins and natural deep eutectic solvents that play a role as alternative media to water. We discuss the chemistry of inter‐organism communication and signalling within the biosphere that allows plants to discriminate between harmful, benign and beneficial microorganisms. We summarize current information concerning the chemical repertoire that underpins plant−microbe communication and host‐range specificity. We highlight how the regulated production, perception and processing of reactive oxygen species (ROS) is used in the communication between plants and microbes and within the communities that shape the soil microbiome.

## Summary statement

Plant−microbe interactions are defined and sustained by a communication highway the involves an extensive repertoire of primary and secondary metabolites. The regulated production of reactive oxygen species (ROS) is an essential component in inter‐organism communication that shapes the soil microbiome and plant−microbe communication.

## 1 Introduction

**Figure 1:**
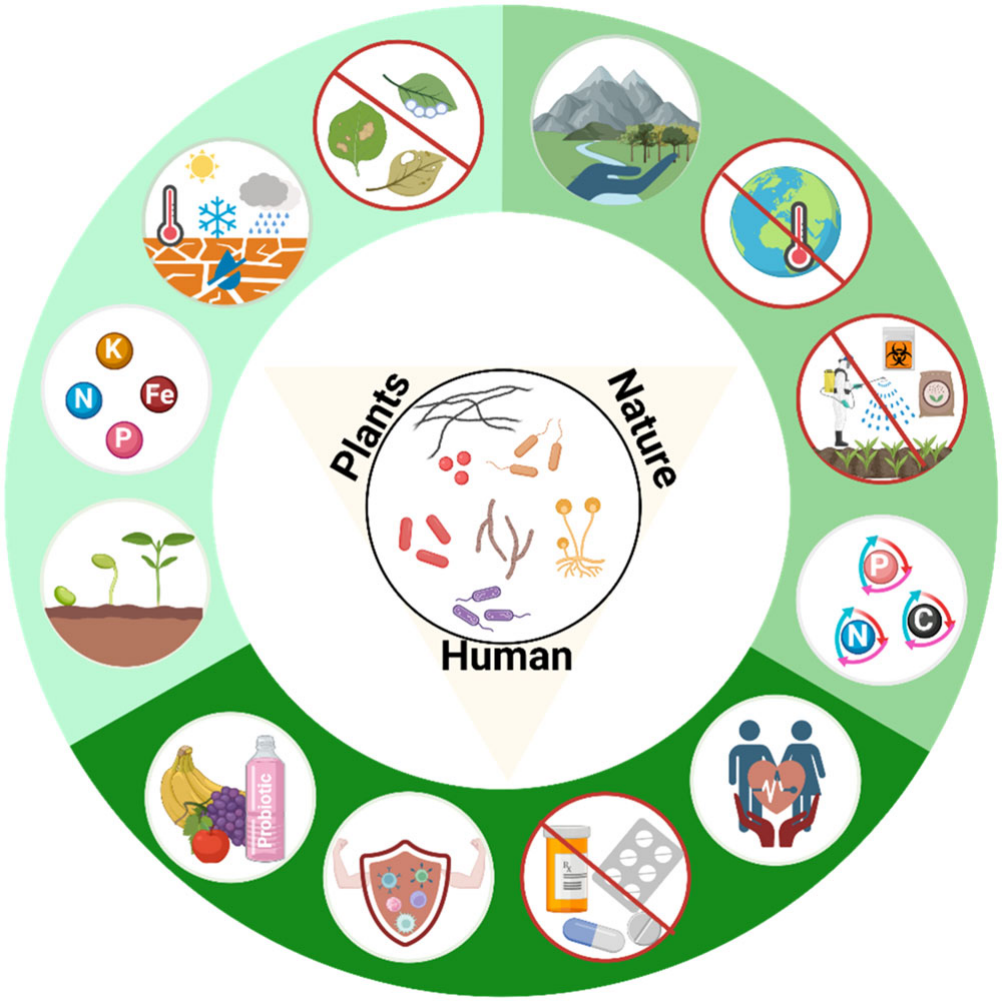
Microbiomes serve as the foundation for one health. The health of plants, humans and the environment are interconnected and rely on close association with microbial communities. The beneficial microbial microbes in the plant rhizosphere determine plant productivity by assisting in seed germination, foraging for nutrients under nutrient‐limiting conditions or nutrient recycling along with providing abiotic and biotic stress tolerance through production metabolites and modulating host immunity, respectively. In humans, the beneficial microbes introduced via direct consumption of plant products or indirect exposure through the environment contribute to overall health maintenance by boosting the immune system against pathogens, reducing the usage of antimicrobial compounds and providing some essential nutrients. The beneficial microbes can offer a sustainable solution for the conservation of nature by replacing chemical fertilizers and pesticides responsible for global warming and environmental pollution.

Holobiome research has rapidly expanded over recent decades. Accumulating literature evidence has validated the holobiont theory, principles of co‐evolution and co‐selection and synergistic stress responses within microbiota and holobionts, particularly plants and their interacting organisms. There is a growing awareness that all eukaryotes, including plants are holobionts and, hence, must be considered together with their microbiota as an inseparable functional unit, particularly within an environmental context. The interconnections between different organisms influence the fitness and performance of the whole ecosystem (Banerjee and van der Heijden 2023). The microbial communities housed within and around plants are considered to function as a ‘second genome’, ‘extended genotype’ or an ‘eco‐holobiont’. Microbial communities influence key functions such as nutrient uptake, host immunity, prevention of pathogen colonization and overall health in systems as diverse as the human gut and plant rhizosphere (Figure 1). Harnessing the power of the second microbial genome to improve food production is therefore not only an attractive target for plant science research but it can also be applied to lower the environmental impact and residual effect of chemicals in the food chain (Trivedi et al. 2022).

Microorganisms are key players in carbon cycling. Microbial carbon metabolism can be more efficient than photosynthesis and encompass processes that convert CO_2_ to methane (CH_4_). Free‐living microorganisms are crucial to the global carbon cycle, not least because they are responsible for about 50% (~120–200 Gt), of annual greenhouse gas (CO_2/_methane [CH_4_]) transformations and contribute to almost 50% of the annual global CO_2_ sequestration. Thus, microbial carbon use efficiency (CUE) is a major determinant of global soil organic carbon storage, surpassing other soil processes. While the molecular mechanism regulating CO_2_/methane interconversions remains poorly understood, this knowledge is required to engineer a more sustainable carbon cycle. The positive correlation between plant−mycorrhiza associations and soil carbon content bears testimony to the importance of nature‐based solutions to combating climate change (Soudzilovskaia et al. 2019). Nitrogen fertilizers are responsible for nearly 5% of emissions of global greenhouse gas mainly nitrous oxide, which is 300 times more potent than CO_2_ in terms of heat‐trapping (Gao and Cabrera Serrenho 2023). Similarly, up to 80% of applied phosphorus fertilizer is lost due to inefficient plant uptake. Excessive nitrogen‐ and phosphorus fertigation can cause soil acidification and fresh‐water eutrophication. Gaining an improved molecular‐mechanistic knowledge of microbe‐environment interactions is one of the biggest challenges in contemporary biology and a prerequisite for viable microbe‐based solutions to climate change.

**Figure 2:**
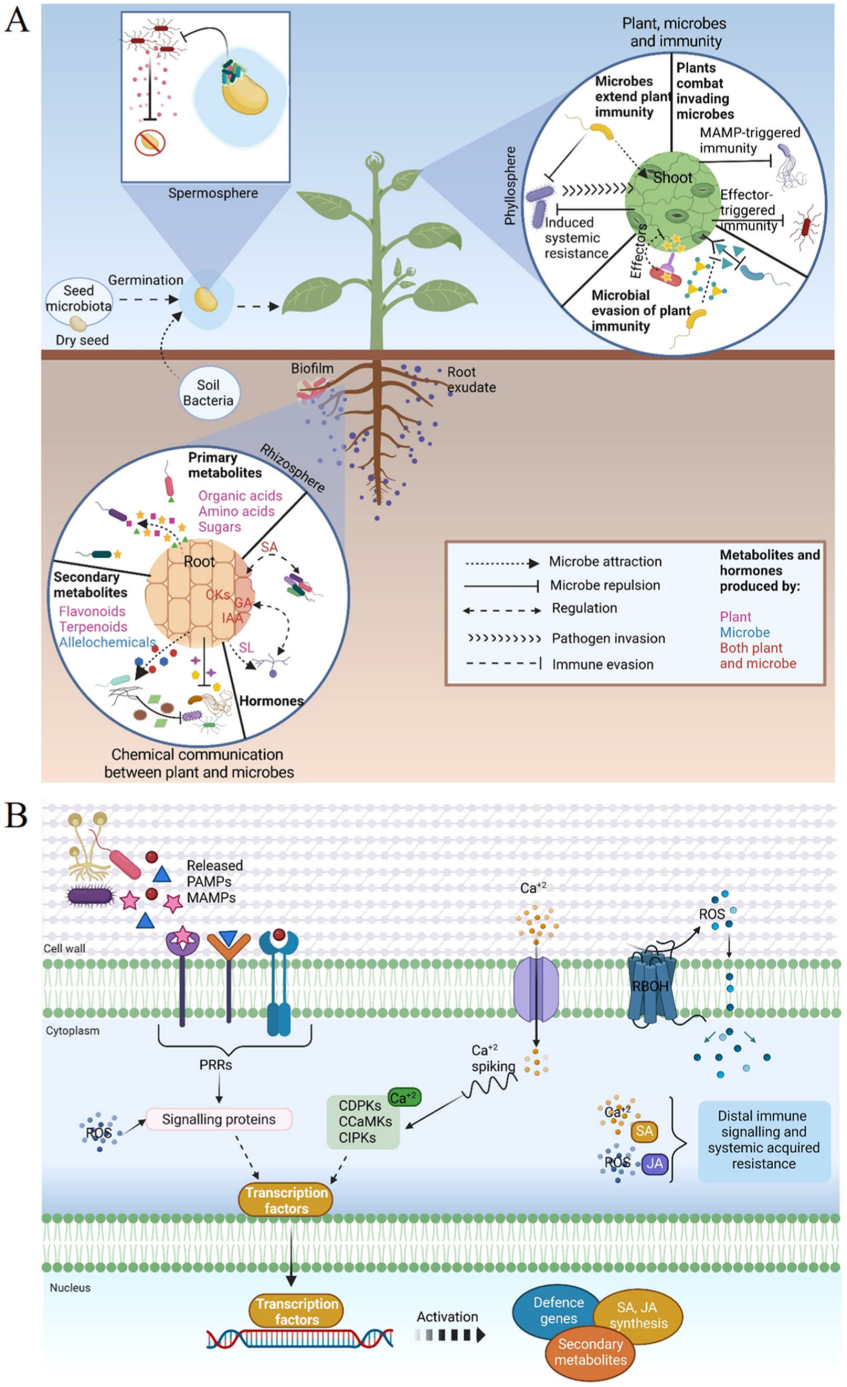
Mechanisms for plant−microbe interactions. Plant−microbe interactions occur in spermoshere, rhizosphere and phyllosphere. Microbes in the spermosphere aid seedling establishment by controlling germination and the secretion of compounds that inhibit competing pathogenic microbes. The plant−microbe chemical communication network involves primary and secondary metabolites that attract beneficial microbes and restrict pathogenic microbes. Beneficial microbes secret allelochemicals with biocontrol activity. Phytohormones including indole acetic acid (IAA), cytokinin (CK), gibberellic acid (GA), strigolactone (SL) and salicylic acid (SA) are secreted by both plants and microbes. Soil microbes not only enhance plant immunity by triggering systemic resistance responses via MAMP‐triggered immunity or effector‐triggered immunity, but they also directly inhibit invading pathogens. Beneficial microbes can successfully evade plant immune surveillance by deploying effector proteins to suppress plant immune response (A). The microbe‐derived PAMPs and MAMPs are perceived by PRRs that trigger signalling events, including Ca influx and activation of plasma membrane‐bound RBOH enzymes that produce reactive oxygen species (ROS burst). Oscillations in calcium concentrations (calcium spiking) and ROS waves act together with a network of signalling proteins as well as Ca‐dependent protein kinases (CDPKs, CCaMKs and CIPKs) to control transcriptional reprogramming, activation of defence‐related genes, secondary metabolite production and defence hormone synthesis. This system triggers an immune response throughout the plant resulting in systemic acquired resistance (B). Current concepts concerning this signalling cascade are described in detail in the accompanying text. [Color figure can be viewed at wileyonlinelibrary.com]

The plant−microbe community includes plant growth‐promoting microorganisms (PGPM) and arbuscular mycorrhizal fungi (AMF) and other endophytes that stimulate plant growth and development under optimal and stress conditions through a range of mechanisms (Figure 2). Endophytes asymptomatically colonize plant tissues (endosphere), as well as living on the outside (ectosphere) of plant organs (Ghatak et al. 2023). Rhizosphere establishment and the persistence of beneficial microbes are dependent on efficient root colonization that facilitates the provision of carbohydrates and other nutrients in root exudes. In turn, microbes assist plants in the uptake of essential micronutrients, particularly nitrogen, phosphorus and potassium. They also produce phytohormones such as indole‐3‐acetic acid (IAA), cytokinins (CK), gibberellins and siderophores. Such microbe‐derived chemicals (biomolecules) are an essential component of the repertoire of inter‐organism communication portfolio that triggers responses that enhance plant fitness and resilience to environmental stresses, such as salt (Li et al. 2021) and drought (de Vries et al. 2020). The vast array of biomolecules that underpin inter‐organism information exchange and their receptors constitute interconnected molecular networks that together form ecological scaffolds, which interweave the metabolic, transcriptomic and proteomic profiles of each interacting organism.

The transgenerational interdependence between plants and their preferred microbiome is carried through the seed microbiota. For example, the epiphytes that inhabit the seed coat and the endophytes that colonize the endosphere (including the embryo, endosperm, aleurone and perisperm) are central to seedling resilience and establishment. The seed microbial community pioneers the next generation not only functions in the seedling establishment but also facilitates subsequent microbial colonization from the spermosphere (Figure 2A).

The concept that microbe‐assisted solutions can improve plant survival from heat waves, floods, droughts and toxic heavy metals is widely accepted. For example, changes in the drought and cold tolerance of tree species have been linked to AMF diversity. Nurturing trees by microbial symbionts acclimatized to specific climatic conditions has been suggested to safeguard their existence by providing climate tolerance (Allsup, George, and Lankau 2023). Similarly, plant−microbe interactions are an indispensable component of ecosystem restoration for example, following excessive use of chemical fertilizers and pesticides.

Many PGPMs support plant growth by increasing nutrient‐use efficiency, biotic/abiotic stress tolerance and disease resistance. Rhizobacteria and free‐living diazotrophs mediate biological nitrogen fixation in nodulating and non‐nodulating crops, respectively. Moreover, PGPMs can secrete organic acids to solubilize insoluble phosphates and, hence, significantly reduce the usage of phosphorus‐fertilizer without jeopardizing crop yield.

To date, most of our current knowledge of the molecular communication that underpins such beneficial interactions is based largely on studies between plants and single bacterial or fungal isolates gained under axenic conditions and we acknowledge that it is therefore challenging to translate this knowledge to field conditions. We therefore focus the following discussion on the molecular dialogue that occurs within interacting fluid matrices and biofilms and defines plant−microbe interactions, while considering the constraints to practical feasibility. We highlight the emerging role of reactive oxygen species (ROS) in quorum sensing and inter‐organism communication, and we consider the importance of other reactive molecules such as nitric oxide (NO). Only when we have gained a complete mechanistic knowledge of the nature of the interactions between the molecules within plant exudates and microbial biofilms will we be able to better understand the bottlenecks and limitations to this communication imposed under field conditions.

## 2 Multiple Pathways for Inter‐Organism Communication

### 2.1 Microbe‐Derived Bioactive Metabolites

Rhizosphere bacteria produce a portfolio of bioactive secondary metabolites and inhibitory allelochemicals that are responsible for induced systemic resistance (ISR), biocontrol and ecological fitness (Figure 2A). For example, the typical plant‐associated species, *Bacillus velezensis* has considerable biocontrol potential based on its ability to trigger ISR leading to rapid and robust defence responses, as well as direct antagonistic actions on pathogens (Ongena et al. 2007). Over 12% of the genome in bacteria such as *B. velezensis* is devoted to the synthesis of compounds that contribute to ecological competence and biocontrol activity. They produce a wide range of non‐ribosomally synthesized compounds such as oligopeptides, cyclic lipopeptides and polyketides, in addition to post‐translationally modified lanthipeptides and bacteriocins. These molecules play a key role in multitrophic interactions because they can be antagonistic with other microbes and/or beneficial to the host via the stimulation of innate immune responses leading to ISR. Bioactive secondary metabolites are not only produced in vitro conditions but also in planta by bacterial cells evolving as biofilm‐structured microcolonies on root tissues (Andrić et al. 2021).

Plant‐associated bacteria have evolved a polymer‐sensing system to perceive the host. They increase the synthesis of bioactive secondary metabolites in response to the perception of root exudates. For example, the production of the cyclic lipopeptide surfactin by *B. velezensis* is stimulated by pectin, which is recognized as a cell surface molecular pattern in root exudates. Surfactin is a bacterial ISR elicitor that can both initiate and inhibit biofilm formation (Stoll et al. 2021). This small signalling molecule regulates quorum sensing responses and is key to plant colonization and biofilm formation on plant surfaces. The synthesis and release of surfactin, which is synthesized non‐ribosomally by a complex enzyme system, is governed by the regulated transcription of the *srfAA‐AD* operon (Qiao et al. 2024). Surfactin, which is formed as a mix of naturally coproduced homologues with fatty acid chains of various lengths optimizes biofilm formation, motility and early root colonization, as well as reinforcing the defensive capacity of the host. The ISR phenotypes triggered by surfactin and other beneficial bacteria are an attractive addition to the crop protection toolbox, as bio‐sourced alternatives to chemicals. However, the molecular basis for the action of surfactin and the wide range of other secondary metabolites produced by PGPMs remains poorly characterized and understood.

### 2.2 Receptor−Ligand Interaction

The complex and reiterative dialogue between plants and soil microorganisms involves extensive local and systemic communication systems that are based, at least in part, on plant receptors that detect microbe‐associated molecular patterns (MAMPs) and pathogen‐associated molecular patterns (PAMPs). Plants employ cell surface‐resident pattern recognition receptors (PRRs) and intracellular nucleotide‐binding domain leucine‐rich repeat (NLR) receptors to detect the presence of microbes. PAMPs and microbe‐generated effectors activate PAMP‐triggered immunity (PTI) and effector‐triggered immunity (ETI), respectively. It appears that each PAMP and effector is perceived by a specific receptor. Pathogen infection leads to the release of different host‐derived damage‐associated molecular patterns (DAMPs) at the infection site that are sensed by PRRs that induce DAMP‐triggered immunity (DTI) (Tanaka and Heil 2021). PTI, ETI and DTI all involve the production of mobile signals in the local tissue that are transported through the vascular system to distal systemic tissues of the plants, triggering the establishment of systemic acquired resistance (SAR). These molecules, which are restricted to pathogens and are not generally conserved and shared by a wide range of microbes, trigger ISR and play a prominent role in determining the success or failure of any plant−microbe interaction (Lü et al. 2022). PTI and ETI mutually potentiate plant immunity, producing a more sustained biphasic oxidate burst than either pathway alone.

Chitin, which is a fungal cell wall homopolymer of unbranched β‐1,4‐linked N‐acetylglucosamine (GlcNAc), is a key MAMP component that is recognized by PRRs (Lü et al. 2022). The breakdown products of chitin (chitooligosaccharides: COs) are perceived by plant lysin motif (LysM) plasma membrane receptors triggering defence‐gene induction, secretion of chitinases, an oxidative burst and restriction of fungal growth. The secretion of LysM‐containing effector proteins that sequester AMF cell‐wall‐derived COs plays a major role in the control of chitin‐triggered host immunity (Zeng et al. 2020). Beneficial microbes, including rhizobia and AMF, which inhabit plant tissues can establish a mutualistic relationship with their hosts by either evading or suppressing host immunity (Figure 2). The molecular dialogue between plant immune system and rhizosphere microbiota not only promotes colonization by beneficial microbes but it also prevents growth‐defence trade‐offs triggered by the MAMP‐rich rhizosphere environment.

Plants secrete a range of chemicals such as flavonoids and strigolactones (SL) that are recognized by symbiotic rhizobia, which in turn secrete bacterial lipo‐chitooligosaccharides (LCOs or Nod factors) that have a core structure of three to five N‐acetyl glucosamines with an acyl chain attached to the nonreducing residue. Rhizobium Nod factors that are perceived by legumes and *Parasponia* species trigger a common symbiotic signalling (SYM) pathway that involves genes that are also required for AMF symbiosis (Escudero‐Martinez et al. 2022; Oyserman et al. 2022). LCO receptor has a well‐conserved hydrophobic structural signature that monitors the composition of amphiphilic LCO molecules. Receptor−ligand interactions provide a kinetic proof‐reading mechanism for the selective recognition‐guided activation of symbiotic signalling in legumes, determining rhizobia‐legume compatibility (Gysel et al. 2021). Rhizobial species either have a broad host range (e.g., *Sinorhizobium fredii* NGR234 interactions with over 200 distantly related legumes) a narrow host range like *S. meliloti* that primarily nodulates *Medicago truncatula* or *M. sativa* (Kelly, Radutoiu, and Stougaard 2017). Many *S. Meliloti* strains induce incompatible signalling due to two *Nodulation Specificity 1* locus (*NS1a* and *NS1b*) genes that encode malectin‐like leucine‐rich repeat receptor kinases, which block tissue invasion and root nodule induction. The activation of the *Nodulation Specificity 1* gene is dependent upon the bacterial gene *rns1* (Root nodule symbiosis) that encodes a Type I‐secreted protein (Liu, Wang, et al. 2022).

Unlike the relatively well‐characterized interactions between plants, AMF and rhizobia, the molecular basis for cooperative interactions between beneficial bacteria and plants remains poorly characterized. Some beneficial bacteria display MAMPs, while others appear to bypass this high‐affinity protein receptor‐mediated surveillance system that triggers host immune responses. These are then recognized by cell‐surface receptors, PRRs. For example, membrane lipid‐dependent elicitation by surfactin induces an atypical early immune response including an intra‐cellular ROS burst and low calcium influx. This atypical response triggers systemic resistance to *Botrytis cinerea* infection in *Arabidopsis thaliana* (Pršić et al. 2023). The surfactin‐induced activation of enhanced immune responses in *A. thaliana* is mediated by docking into specific sphingolipid‐enriched domains leading to host membrane deformation and activation of mechanosensitive ion channels (Pršić et al. 2023).

### 2.3 Redox‐Mediated Signalling

An intrinsic feature of both PAMP and MAMP triggered responses, as well as physical and chemical perturbations to the plant plasma membrane, is the propagation of ROS and cytoplasmic calcium ([Ca^2+^]_cyt_) waves from the cell perceiving the initial signal to all cells throughout the plant (Figure 2B). Together with fluctuations in electric wave potentials, the Ca^2+^_cyt_/ROS waves trigger long‐distance systemic defence signalling cascades that induce ISR throughout the plant. The ROS wave is not only an essential systemic signalling system in plants, but it is also important in plant‐to‐plant communication (Szechyńska‐Hebda et al. 2022) and in plant−microorganism interactions (Peláez‐Vico et al. 2024). Changes in the levels of apoplastic ROS are perceived by specific receptors on the plant plasma membrane. While research on plant ROS receptors in plants is at a very early stage, the leucine‐rich repeat receptor‐like kinase called Hydrogen Peroxide Ca^2+^ Increases 1 (HPCA1) is a key apoplastic ROS receptor. Perception of oxidation in the apoplast activates the HPCA1 kinase, triggering the transmission of MAPkinase signalling associated stress responses within the cell. The HPCA1 kinase is also required for the transmission of cell‐to‐cell signals that activate systemic acclimation responses to environmental stresses (Fichman et al. 2022). ROS wave signalling is not limited to plants but is found a wide range of microbial cell communities and in mammals (Peláez‐Vico et al. 2024). ROS wave‐based cell‐to‐cell communication is likely to have evolved as a type of stress‐ or quorum‐sensing. ROS signals are an essential component of the communication exchange between different organisms within a given environment.

The ROS wave is triggered by an oxidative burst in the apoplast, which occurs because of the Ca^2+^‐dependent activation of plasma membrane‐bound NADPH oxidases (Respiratory Burst Oxidase Homolgues: RBOH) and cell wall peroxidases. These enzymes generate superoxide in the apoplastic space, that is, the external space around the cell walls, which stores water and nutrients. The oxidative burst is one of the earliest plant responses to infection by microbes such as endophytes (Sahu et al. 2022). The apoplast has a low antioxidant capacity, but it is rich in antifreeze proteins and pathogen‐related proteins (PR‐proteins) such as proteinase inhibitors, defensins, thionins and lipid transfer proteins that together represent 23%−33% of the total apoplastic fluid proteins (Farvardin et al. 2020). Localized ROS accumulation in the apoplast and surrounding fluids can create micron‐scale membrane‐less compartments formed by liquid‐liquid phase separation. This type of regulation may contribute to signal perception and processing by as yet uncharacterized pathways.

The perception of pathogens triggers prolonged activation of RBOH and other ROS‐producing enzymes leading to the hypersensitive response (HR) that prevents the spread of the pathogen from the attacked cell to neighbouring cells. ROS plays a central role in the signalling pathways that underpin SAR. The oxidative burst at the site of infection is one of the earliest cellular responses following pathogen infection that can spread to systemic tissues. Many molecules, including ROS and NO have been identified as potential mobile signals that play essential roles in SAR. NO and related NO‐mediated S‐nitrosylation regulate ROS production, to enhance the ROS burst and disease resistance (Liu, Liu, and Mou 2024). Avirulent pathogens trigger biphasic oxidative and nitrosative bursts that have a low‐amplitude, transient first phase associated with PTI, followed by a sustained phase of greater ROS production resulting from the coordinated action of both PTI and ETI. A pathogen‐induced phosphatidic acid (PA) burst that is mediated by the PRR‐associated kinase Botrytis‐induced kinase 1 (BIK1)‐mediated phosphorylation of diacylglycerol kinase 5 (DGK5) triggers ROS accumulation during PTI and ETI (Kong et al. 2024). DGK5 is subsequently phosphorylated by PRR‐activated MPK4, resulting in suppression of DGK5 activity and PA production. Virulent pathogens only induce the initial transient, low‐amplitude phase of ROS accumulation that is not associated with disease resistance. Some virulent pathogens use effectors or virulence factors, such as the *Pseudomonas syringae* virulence factor coronatine to suppress PTI‐associated ROS production. Coronatine suppresses early PTI‐associated ROS accumulation in the cytosol and induces ROS production in chloroplasts at the later stages of infection (Liu and Zhang 2022). The extent and duration of ROS production, together with the intracellular and extracellular localization of ROS accumulation are therefore crucial to the outcome of plant−microbe interactions.

Endophyte‐triggered ROS generation does not lead to HR but in contrast, triggers systemic signalling leading to defence gene expression (Sahu et al. 2022) and wide‐ranging enhanced protection against biotic and abiotic stresses (Godara and Ramakrishna 2023). The oxidative burst facilitates the oxidation of secondary compounds in the apoplast/cell wall environment, such as the conversion of sesquiterpenoids to oxygenous sesquiterpenoids through chemical oxidation and differing degrees of oxidation. Endophytes also activate the expression of genes encoding key enzymes involved in secondary metabolism, such as those involved in sesquiterpenoid biosynthesis, creating an array of sesquiterpenoid hydrocarbon scaffolds (Zhou et al. 2016). For example, the microbe‐induced oxidative burst and oxygenous sesquiterpenoid accumulation occur synchronously upon colonization of the medicinal plant *Atractylodeslancea* by *Pseudomonas fluorescens* ALEB7B. Sesquiterpenoids such as hinesol, β‐eudesmol, atractylone and caryophyllene oxide have medicinal properties protecting against rheumatic diseases, digestive disorders, night blindness and influenza (Zhou et al. 2016).

### 2.4 Phytohormone‐Based Signalling

The pre‐symbiotic chemical dialogue between fungus and host plants is the essential first step in the path to successful plant−microbe interactions. Phytohormones such as SLs and karrikins (KARs) condition pre‐symbiosis rhizosphere communication with AMFs. *A. thaliana* (a non‐mycorrhizal plant) SLs and KARs receptors D14 (Dwarf14) and KAI2 (Karrikin‐insensitive 2; rice homologue of D14L) activate downstream signalling through the MAX2 (More axillary growth 2) E3 ligase. MAX2 targets different members of the SMAX1‐LIKE (Suppressor of MAX2‐1‐like) family of transcriptional repressors for degradation. The *d14l* and *d3* mutants show impaired AMF colonization (Gutjahr et al. 2015). Perception of AMF by the rice α/β‐fold hydrolase D14L (Dwarf14‐Like; an evolutionary paralogue of D14) leads to D3 (Dwarf3; a homologue of MAX2)‐mediated signalling, stimulating fungal metabolism, allowing rapid hyphal growth in roots and expanding colonization. KAI2 functions are conserved in monocots (Meng et al. 2022). SMAX1 operates downstream of the D14L/D3 receptor to negatively regulate AMF symbiosis. Rice *smax1* mutants show enhanced expression of SL biosynthesis genes, indicating that SL‐KAR crosstalk is required for efficient AMF symbiosis (Choi et al. 2020). AP2/ERF transcription factors (TFs), such as the ERM1 and ERF12 TFs in *M. truncatula* are also important in establishing a symbiotic relationship between AMF and host plants. These TFs are significantly expressed in mycorrhizal roots. While ERM1 positively regulates arbuscule development by activating the expression of lipid synthesis and transport genes, ERF12 acts as a negative transcriptional repressor (Zhang et al. 2023).

Gibberellic acid (GA) and the associated GRAS family DELLA TFs mediate transcriptional reprogramming during arbuscule development. GA binding to its receptor induces the degradation of DELLA proteins that repress GA signalling. The *della* mutants of *M. truncatula* (Floss et al. 2016) and rice (Yu et al. 2014) exhibit impaired AMF symbiosis. Similarly, the exogenous application of GA inhibited arbuscule branching in pea roots (El Ghachtouli et al. 1996). Expression of the MIG1 (Mycorrhiza‐induced GRAS1) GRAS‐type TF, which is a key regulator of cortical cell expansion, is a prerequisite for arbuscule development. The expression of dominant‐active DELLA proteins rescues the loss‐of‐arbuscule phenotype of the *mig1* mutants. Hence, MIG1 and DELLA act together to promote cortical radial cell expansion (Heck et al. 2016). The MIG1‐DELLA‐mediated positive regulation cascade is fine‐tuned by MIG3 (a MIG1 paralogue)‐SCL3 (Scarecrow‐like 3, GRAS TF), which acts as a negative regulator of arbuscule development in *M. truncatula* (Seemann et al. 2022). Salicylic acid (SA) regulates root−microbiome interactions in response to nutrient deficiency (Kim et al. 2022). SA signalling pathway is also central to the establishment of SAR (Gao et al. 2015). NO and ROS‐mediated redox signalling pathways regulate SA synthesis and the activation of the SAR transcription regulators NONEXPRESSOR OF PATHOGENESIS‐RELATED (PR) GENES1 (NPR1) and TGACG motif‐binding (TGA) TFs (Liu, Liu, and Mou 2024). In turn, SA induces both RBOH‐dependent and peroxidase‐dependent ROS production, while also inhibiting the action of enzymes such as catalase and cytosolic ascorbate peroxidase that remove hydrogen peroxide. In addition, SA induces the formation of an NPR1‐Cullin E3 ligase complex to ubiquitinate ROS‐scavenging proteins, which probably induces their degradation. Via the regulation of ROS, SA is considered to orchestrate a complex array of posttranslational modifications of downstream signalling components, as well as promoting the formation of biomolecular condensates that function as cellular signalling hubs. In this way, SA may coordinate the whole phytohormone signalling network.

Rhizobia‐ and AMF‐induced microbial signals are transduced via nuclear‐localized calcium oscillations, generated by CNGC15 (Cyclic‐nucleotide gated channel 15). The calcium‐bound form of calmodulin 2 (holo‐CaM2) is required to sustain prolonged CNGC15‐dependent calcium oscillations. An engineered holo‐CaM2 enhanced root nodule symbiosis but not arbuscular mycorrhization, indicating that the holo‐CaM2‐CNGC15s module is specific to Rhizobial symbiosis (Del Cerro et al. 2022). Calcium‐phytohormone crosstalk functions alongside bidirectional nutrient exchange to establish rhizobia/AMF symbioses.

The light‐induced GmSTF3/4 (soybean TGACG‐motif binding factor 3/4) and FLOWERING LOCUS T (GmFTs) proteins function as mobile shoot‐to‐root signals. CCaMK (calcium‐ and calmodulin‐dependent protein kinase) mediated phosphorylation of GmSTF3 triggers the formation of a GmSTF3−GmFT2a complex, which activates the expression of nodule inception (NIN) and nuclear factor Y (NF‐YA1 and NF‐YB1) that together regulate nodule organogenesis. The CCaMK–STF–FT module ensures a sustained shoot carbon supply (Wang, Guo, et al. 2021). Rhizobial colonization, which is impaired in *della* mutants, is tightly controlled by host‐derived GA signals. DELLA proteins promote the formation of the CCaMK–IPD3 complex. They also interact with NSP2 (Nodulation Signalling Pathway2), enhancing the expression of Nod‐factor‐inducible genes that are required for NSP1 binding to the NIN promoter (Jin et al. 2016). In this way, DELLA integration of the IPD3 and NSP1/2 pathways provides the transcriptional framework for successful root nodulation.

### 2.5 Bidirectional Nutrient Exchange

The requirement for essential soil nutrients, such as nitrogen (N) and phosphate (P), is another major driver for symbiotic associations between plants and beneficial microorganisms, including AMFs and nitrogen‐fixing rhizobia. AMF establishes root symbiosis with more than 80% of terrestrial plants (Chan 2022). The partnership is mutually beneficial but not exclusive that is, plants can form associations with multiple microorganisms simultaneously. Such associations assist plants in the acquisition of water and essential minerals, particularly P and N. In return, AMF and other microorganisms receive assimilated carbon. AMF‐mediated N/P uptake pathways function alongside the plant nutrient transport systems, providing a greatly expanded network of nutrient uptake from distal regions beyond the normal reach of roots or root hairs.

Plants control the AMF symbioses to the availability of orthophosphate (Pi), the preferred form of P taken up by roots (Lu et al. 2022). The plant Pi sensing SYG1/Pho81/XPR1 (SPX)—PHOSPHATE STARVATION RESPONSE (PHR) pathway regulates AMF symbiosis in crops such as rice (Shi et al. 2021), *Medicago* (Li et al. 2021) and tomato (Liao et al. 2022). The SPXs Pi sensing proteins regulate the activity of the PHR family of R2R3 MYB TF. Many genes required for AMF symbiosis have PHR1‐binding sites (P1BS) and are activated by PHR binding. SPX negatively regulates PHR in rice and tomato, and hence, suppresses AMF symbiosis under moderate‐Pi or Pi‐replete conditions. In contrast, the *Medicago* MtSPX1 and MtSPX3 proteins are positive regulators of AMF colonization through the regulation of SLs biosynthesis. Thus, there appears to be a diversification of SPX functions between cereals and legumes (Wang, Snijders, et al. 2021).

The activation of a subset of Pi‐starvation induced or phosphate‐starvation response (PSR) genes, is a functional marker of successful AMF symbiosis. However, the genetic predisposition of the host plant is a major determinant of the efficiency of AMF symbiosis, as observed in maize accessions (Sawers et al. 2017). PHR2 (Phosphate Starvation Response 2) is a major TF regulating PSR in rice. It promotes AMF colonization by activating pre‐contact signalling genes and mediates mycorrhizal Pi uptake (Das et al. 2022). The *OsADK1* (Arbuscule Development Kinase 1) receptor‐like kinase, which is required for mycorrhizal colonization and arbuscule development, is an OsPHR2 target (Shi et al. 2022). The *Rhizophagus irregularis* SPX‐domain containing PTs regulates arbuscule development and fine‐tunes symbiotic Pi transfer ‐to the plant (Xie et al. 2022). Similarly, the *Gigaspora margarita* high‐affinity PT, GigmPT transceptor activates a protein kinase A‐mediated signalling cascade leading to Pi transport (Xie et al. 2016). Other AMF‐activated genes, such as *LjPT4* and *MtPT4* also serve Pi sensing functions in *L. japonicus* and *M. truncatula*, respectively (Volpe et al. 2016). The extraradical AMF also transports Pi‐solubilizing bacteria (PSB) that enhance organic P mineralization and increase Pi delivery to plants, via AMF‐independent pathways (Jiang et al. 2021).

N‐availability regulates plant‐AMF symbiosis through proteins such as OsNPF4.5. Knockout lines lacking OsNPF4.5 functions had fewer arbuscules and lower (45%) symbiotic nitrate uptake (Wang et al. 2020). The lipids and carbohydrates in root exudates support fungal growth (Kameoka and Gutjahr 2022). Lipid provision involves two lipid biosynthetic enzymes (FatM and RAM2) and two ABC transporters (STR1 and 2; Stunted Arbuscule 1 and 2). FatM (An acyl‐ACP thioesterase) increases plastid export of 16:0 fatty acids. Thereafter, RAM2 (Required for Arbuscular Mycorrhization 2) produces 16:0 b‐monoacylglycerol, which is exported from the root cells across the peri‐arbuscular membrane (Bravo et al. 2017). The *M. truncatula* AP2/ERF family TF MtWRI5a binds to *STR* and other AMF‐specific gene promoters (such as *MtPT4*) to promote lipid and Pi transport from host‐to‐AMF and AMF‐to‐host, respectively (Jiang et al. 2018). The CBX1 (CTTC Motif‐Binding Transcription Factor 1; a WRI1 homologue), which is enriched in AMF‐regulated genes, activates *L. japonicus LjPT4* and lipid‐metabolism genes (Xue et al. 2018). The rice *Osadk1*‐defective mutants have low expression of *RAM1* and *WRI5* and produce fewer arbuscules (Shi et al. 2022). These findings suggest that MtWRI5a, LjCBX1 and OsADK1 are master regulators of bidirectional nutrient transport.

## 3 Environmental Stress Tolerance

Plant‐associated microbial communities are known to impart tolerance against salt (Li et al. 2021) and drought (de Vries et al. 2020) stress conditions. Salt‐induced decreases in root nodulation (Singh and Valdés‐López 2023) involve the expression of a glycogen synthase kinase 3 (GSK3)‐like kinase, GmSK2‐8, which phosphorylates GmNSP1a, preventing the binding of symbiotic genes such as *GmNINa* and reducing nodulation (He et al. 2021). The salt‐inducible GmNAC181 TF activates *GmNINa* expression and maintains soybean nodulation under salt stress (Wang et al. 2022). Drought triggers a compartment‐specific restructuring of rice root microbiota in rice, particularly in the endosphere. Drought increases the prevalence of monoderm bacteria, which lack an outer cell membrane and contain thick cell walls (Santos‐Medellín et al. 2021; Xu et al. 2018). Apart from providing drought adaptation, the microbial communities also contribute to drought‐induced stress memory in rice, through compositional shifts that help plants to survive better upon rewatering (Santos‐Medellín et al. 2021). In the bioenergy model grass, *Panicum hallii*, AMF inoculation under water‐limiting conditions has been shown to impart resistance to sensitive bacterial communities in the hydrosphere, indicating fungal‐bacterial synergy (Hestrin et al. 2022). However, these findings need more careful investigation, while designing microbe‐based solutions for realistic field scenarios. For example, drought‐induced increased prevalence of beneficial microbes in rhizosheaths has been demonstrated to induce risk factors for harmful fungi (Lei et al. 2023).

In addition to nutrient limitation, other environmental stresses have a profound effect on innate immunity, which contributes to the recruitment of beneficial microbes. Pi‐starvation induces the PHR1 TF, which activates the expression of *RALF* (Rapid alkalinization factor) genes. RALF peptides hijack the FERONIA kinase to suppress plant immunity and allow colonization by specialized root microbiota such as *P. fluorescens* (Tang et al. 2022). FERONIA inhibitors, including reversine and staurosporine, enhance innate immunity against soil‐borne diseases in tobacco, tomato and rice without imposing growth penalties (Liu, Li, et al. 2023). *Feronia* mutants show low ROS accumulation and harbour elevated levels of rhizosphere pseudomonads, suggesting that FERONIA participates in the discrimination of beneficial and harmful microbes (Song, Wilson, et al. 2021). Additionally, the water‐deficit conditions release flavonoids that reshape the root microbiome by attracting *Aeromonas* species that enhance dehydration resistance in plants (He et al. 2022). In addition to plant exudates, the core bacterial commensals and host tryptophan‐derived specialized metabolites also function to control the overgrowth of fungal species (Wolinska et al. 2021), thereby maintaining host−microbe as well as microbe−microbe homoeostasis.

## 4 Broadening Host‐Range Specificity for Producing ‘Greener’ Climate

**Figure 3:**
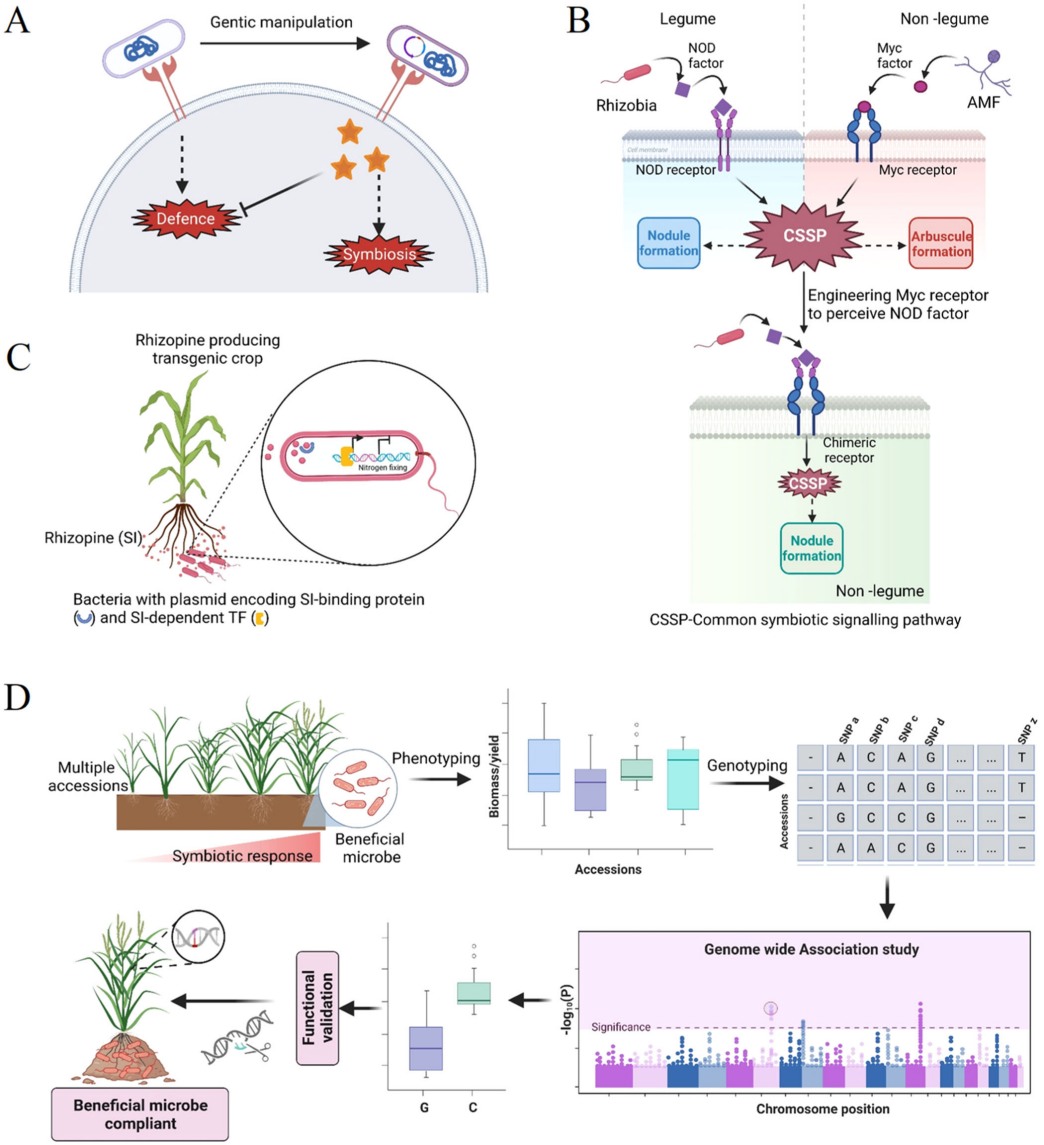
Genetic approaches to broaden the host range of beneficial microbes. Microbe‐derived effector proteins can be engineered to block host defences and initiate symbiosis (A). Genetic engineering approach to recreate nodule organogenesis in nonlegume plants. The Myc‐receptor, which recognizes AMF‐produced Myc‐factors, has been engineered to perceive Nod factors by replacing the outer domain of the receptor. This induces the formation of nodule‐like structures by activating a common symbiotic signalling pathway (CSSP) (B). Another approach is to facilitate the association between nitrogen‐fixing microbes and cereals including rhizopine synthesis in plants such as wheat and rice. Rhizopine is sensed by nitrogen‐fixing bacteria via biosensor plasmids that encode rhizopine‐binding proteins and rhizopine‐dependent transcription factors (TF). These proteins drive the downstream expression of genes encoding proteins required for nitrogen fixation (C). Finally, the recruitment of root‐associated microbiota is dependent on host genetics. Genome‐wide association study mapping will identify novel host genetic loci that control microbial selection to facilitate breeding efforts to improve crop resilience (D). [Color figure can be viewed at wileyonlinelibrary.com]

The increasing levels of CO_2_ in the atmosphere drive photosynthesis and biomass accumulation in C3 crops (Ainsworth and Long 2021). This CO_2_ fertigation effect increases plant growth but negatively impacts crop nutritional quality (Myers et al. 2014) with marked reductions in N, P and other essential mineral nutrients (McGrath and Lobell 2013). The increasing requirement for essential soil nutrients is an important driver for improving symbiotic associations between plants and beneficial microorganisms (Chan 2022). Multiple approaches are underway to broaden host‐range specificity (Figure 3) and to minimize high CO_2_‐induced decreases in crop quality. Of these, altering and/or suppressing host‐induced immunity is perhaps the most important. For example, the downstream target of MtRAM1, *MtKIN3*, suppresses host defences and supports AMF symbiosis (Irving et al. 2022), while also regulating plant N responses. Rhizobia produces several effectors that manipulate host defence signalling pathways. For example, the soybean Nodulation outer protein T (NopT, an effector protease from *Sinorhizobium* sp.) activates the GmPBS1‐mediated resistance pathway and impairs nodule formation (Khan et al. 2022). Similarly, the GmNNL1 (Nodule Number Locus 1; an R protein) protein directly interacts with the NopP effector from *Bradyrhizobium* USDA110 to trigger immunity and inhibit nodulation (Zhang et al. 2021). Thus, genetic engineering of effector proteins to block defence but support symbiosis could broaden the range of beneficial plant−microbe interaction (Figure 3A). Dual‐sensing receptors can widen the host‐range of beneficial microbes (Figure 3B). For example, the expression of chimeric ‘Nod‐Myc’ receptor in which the ectodomains of OsMYR1 and OsCERK1 were replaced by homologous *M. truncatula* sequences in rice led to increased Nod factor‐induced calcium oscillations (He et al. 2019). Similarly, the binding affinity of the *L. Japonicas* receptor kinaseEPR3a (Exopolysaccharide receptor 3a) that binds AMF‐specific glucans, as well as with rhizobia‐specific exopolysaccharide (EPS) could function as a dual receptor. In addition, trans‐kingdom signalling can also be exploited to enhance interactions with beneficial microbes (Soudzilovskaia et al. 2019). For example, barley lines expressing the plant‐derived signal rhizopine, which controls the N_2_‐fixation‐related gene expression in bacteria (Figure 3C), can associate with *Azorhizobium caulinodans*, which has a rhizopine uptake system and usually forms a nitrogen‐fixing symbiosis with *Sesbania*.

For any PGPM, it is crucial to colonize as well as sustain in the dynamic environment also called rhizo‐competence which in part depends on host genetics. Identification of intraspecific variation controlling microbial selection and shaping root‐associated microbiomes remains challenging (Zboralski et al. 2022). Nevertheless, genome‐wide association studies (GWAS) appear to be a potentially powerful tool in identifying host genetic loci which are microbes responsive (Figure 3D). To explore this, previous efforts have demonstrated, the dependence of rhizosphere microbial communities on distinct genotypes of the same host species, including maize and sorghum. Moreover, in *A. thaliana* host SNPs controlling defence and cell wall integrity affected microbial community variation (Deng et al. 2021). Such precision‐based microbiome management in the future could assist in engineering high‐yielding/climate‐resilient crops.

## 5 Engineering Plant−Microbe Interactions

**Figure 4:**
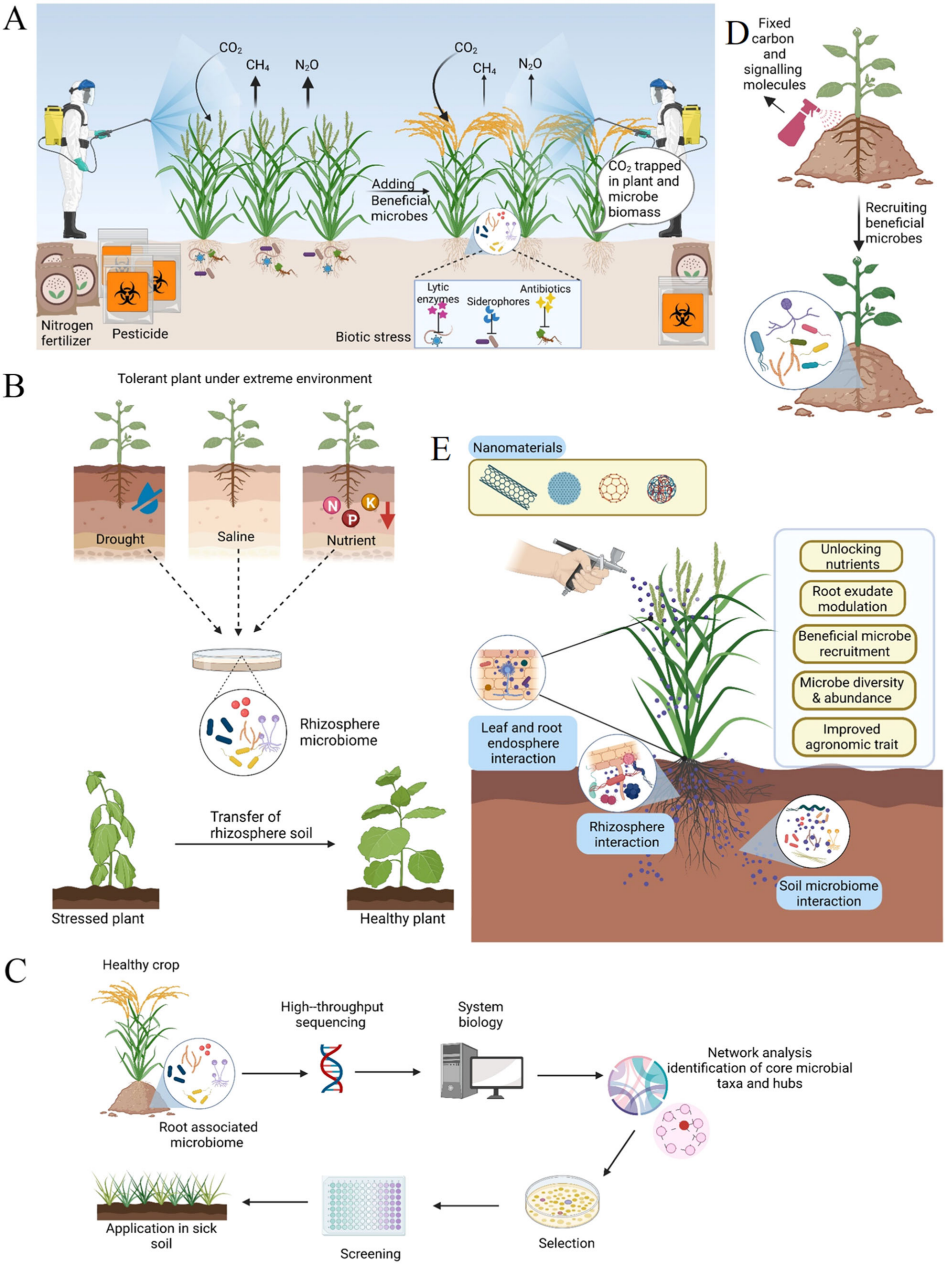
Holobiont approaches for next‐generation agriculture. Beneficial microbes can reduce the agricultural use of fertilizers and chemical pesticides, and contribute to rhizosphere carbon sink capacity (A). Rhizosphere microbiomes from plants inhabiting extreme environments or stress‐tolerant cultivars and can be transplanted can be used in phytoremediation and soil improvement approaches (B). Synthetic microbial communities (SynCom) can augment crop quality. High‐quality crop production systems can be used to source SynComs. High‐throughput sequencing and systems biology approaches can be used to identify appropriate SynCom systems. Network analysis provides information on core microbial taxa and hubs and facilitates the selection of best candidate microbes for further screening. The efficacy and ecological impacts of SynComs must be validated in field studies (C). Composites of the signalling chemicals found in root exudates, including flavonoids, γ‐aminobutyric acid, malate and citrate can be applied to soils to attract beneficial microbes (D). Nanomaterial‐based approaches are also an attractive approach for improving plant health by releasing trapped soil nutrients and modulating root exudates (E). [Color figure can be viewed at wileyonlinelibrary.com]

Several excellent recent reviews, such as Saad, Eida, and Hirt (2020); Ke, Wang, and Yoshikuni (2021); and Banerjee and van der Heijden (2023) have discussed the considerable barriers that currently prevent the effective translation of lab‐based and greenhouse experiments into the field. The root‐associated microbiome is shaped by many factors under field conditions including the substrate (soil), agricultural management practices, the indigenous plant microbiome, plant species and genotype, plant developmental stage and secreted metabolites, soil type, tillage, fertigation, pre‐crops and inoculants (Quiza et al. 2023). While there is considerable potential for PGPMs to serve as biological alternatives to these pesticides and fertilizers (Figure 4A), and also provide abiotic stress tolerance (Mitter et al. 2021), tailoring the soil microbiome to boost plant yield is currently limited by a lack of a full understanding of the global complexity of plant−microbe communication. Additionally, the Anna Karenina Principle, which considers that dysbiotic microbiomes are intrinsically different, while healthy microbiota is similar (Arnault, Mony, and Vandenkoornhuyse 2023), needs to be addressed for using microbial solutions for enhancing crop production and/or soil carbon sequestration with lower fertilizer/pesticides inputs (Figure 4A).

Rhizosphere microbiome transplantation (RMT), in which microbial communities from either extreme environments or from a tolerant cultivar are inoculated to enhance growth or suppress stress responses (Figure 4B) has been used to enhance crop production (Poppeliers, Sánchez‐Gil, and de Jonge 2023). Although some progress has been made under controlled environmental conditions, considerable progress is still required concerning improved agricultural practices, optimizing probiotic microbial consortia and the development of new plant varieties that have improved microbial associations. Besides, soil microbial communities are not constant and vary according to developmental stage, tissue type and sampling time (Quiza et al. 2023), which provides another layer of complexity towards successful RMT. Moreover, the phyllosphere is a conducive niche for horizontal gene transfer (HGT) between epiphytic bacterial strains. The plant surface thus provides a niche for the evolution of new variants. Gaining a deeper understanding of this dynamic environment for gene exchange and the emergence of new strains, together with the exploitation of the remarkable genomic diversity within epiphytic populations, will help overcome current limitations in host‐range specificity and allow potential jumping to other host plants.

Much attention has also focussed on engineering nitrogen‐fixing nodulation traits in nonleguminous crop plants (Huisman and Geurts 2020). However, environmental perturbations exert effects on the diversity of plant microbial communities, with varying effects depending on the plant species and developmental stage. For example, drought increases the release of flavonoids in root exudates. These reshape the root microbiome by attracting *Aeromonas* species that enhance dehydration resistance in plants (He et al. 2022). In addition, core bacterial commensals and host tryptophan‐derived specialized metabolites participate in the control of fungal species (Wolinska et al. 2021). The changes in the relative abundance of Actinobacteria and Proteobacteria were reported in rice cultivated under drought conditions, an effect that persisted after stress alleviation (Santos‐Medellín et al. 2021). In sorghum, drought increased the abundance of *Actinobacteria* and decreased pathogenic genera (*Fusarium, Gibberella and Sarocladium*) compared with well‐watered controls (Gao et al. 2020; Xu et al. 2018). Drought‐stressed sorghum plants in soils with *Arthrobacter* bacteria suffered more than those in which bacteria of the genus *Variovorax* were abundant (Qi et al. 2022). In contrast, *Arthrobacter* alleviated drought‐stress effects in wheat (Hone et al. 2021). The presence of endospheric *Streptomyces* was correlated with drought tolerance in several plant species (Fitzpatrick et al. 2018). Plants grown in natural‐ and agro‐ecosystems preferentially recruit beneficial bacteria and AMF in drought situations (Song and Haney 2021; Zhao et al. 2023). While commercial AMF inoculants, added as a supplement to agricultural lands, have the potential to alleviate stress (Salomon et al. 2022), the diverse nature of AMF functionality must be understood, particularly regarding environmental effects. Microbial consortia may be more efficient than single‐strain inocula in enhancing stress tolerance (Bradáčová et al. 2019). While the AMF richness of barley roots increased under drought conditions, AMF performance (colonization and the abundance of arbuscules and vesicles) was decreased, indicating antagonistic interactions (Sendek et al. 2019). Moreover, while drought increased the prevalence of beneficial microbes in the rhizosheath, it also increased the risk of penetration by harmful fungi (Lei et al. 2023).

Native synthetic microbial communities (SynComs) from plants grown under optimal conditions can be used to boost plant growth in poor soils (Figure 4C) (Jiang et al. 2023). For example, SynComs application promotes the growth of *A. thaliana*, in an innate immunity‐dependent manner (Wolinska et al. 2021). Cross‐kingdom (fungi and bacteria) SynComs were more effective in suppressing fusarium‐wilt disease in tomatoes than fungi or bacteria alone (Zhou et al. 2022). RMT and SynComs have yet to be tested for field applications, due to low efficiency in isolating functionally beneficial microbiomes. Multiple approaches based on screening natural variation, mathematical modelling, RAMAN‐spectra and successive passaging are being used to map the microbial community networks (He et al. 2021). The root‐secreted chemicals or exudates comprised of photosynthetically fixed carbon as well as diverse signalling molecules, including γ‐aminobutyric acid, malate and citrate, also contribute to shaping the rhizosphere microbiome (Figure 4D). In addition, microbe‐derived secretory proteins YukE cause iron leakage in plant roots, which contributes to root colonization by beneficial rhizobacterium *B. velezensis* (Liu, Shu, et al. 2023). The QTLs that determine tomato root‐microbiota composition contain genes such as the iron regulator FIT and water channel aquaporin SlTIP2.3 (Oyserman et al. 2022) and NLR in barley (Escudero‐Martinez et al. 2022).

Nano‐enabled approaches have the potential to overcome the limitations of traditional microbiome engineering, such as the absence of specificity in attaining targeted manipulation, collateral fatality to microbial diversity and lack of reliable robust results (Figure 4E) (Hussain et al. 2023). However, the application of novel nanomaterials still requires comprehensive profiling to evaluate long‐term efficacy on plant productivity and ecosystem health. The beneficial actions of such approaches have been studied largely under controlled conditions and, hence, effective translation into the field is uncertain. The practical relevance of increasing mechanistic knowledge hence requires extensive study under field conditions (Kaminsky et al. 2019). Such information is essential to understand how we might best apply inoculants to fields, which remains a significant challenge.

## 6 Conclusions and Perspectives

Cell signalling plays a vital role in the perception of any microbial shifts, enabling individual cells to execute appropriate biological responses. Additionally, the specificity and fidelity of signalling molecules are core to information exchange between different organisms within a given environment. They facilitate the coordination of complex processes that either accept or reject the advances of different interacting organisms, allowing symbiosis while rejecting potentially harmful or less beneficial relationships. Although our current understanding of the discrete signal transduction pathways in individual cell types is advanced, many gaps remain in our knowledge. For example, we know relatively little about how cells identify and distinguish the array of input signals from other cell types and translate them into distinct outcomes. Similarly, the interactions between and within the rhizosphere liquids (exudates, biofilms) are poorly understood. There is also the possibility that metabolites that occur in large amounts in such liquids may form a third type of liquid called natural deep eutectic solvents as an alternative medium to water (Choi et al. 2011). Such information bottlenecks must be overcome so that plant and microbial communities can be tailored to realize maximum benefits in crop production and/or carbon sequestration.

Various mechanisms, including microbe‐derived metabolites, receptor−ligand interactions, redox‐mediated signalling, phytohormone‐based signalling and bidirectional nutrient exchange have been identified in specific microbe−plant systems. However, it remains debatable whether these insights can be generalized to all members of plant microbiomes. Furthermore, plants have a decentralized organizational structure in which the signal integration and decision‐making processes is distributed between different cell types and organs. Within this context, ROS production/accumulation in the plant apoplast and also by rhizosphere microbes presents an intriguing communication highway that connects quorum sensing to systemic signalling within and between different cell types within a community. As yet, little is known about how the cells of different interacting organisms perceive and translate ROS signals within the context of inter‐organism communication. Thus, we propose that the individual responses of different cell types to the perception of different signalling molecules in terms of localized inter‐ and intra‐cellular ROS production and signalling is intrinsic to inter‐organism communication. We consider that together with NO and reactive nitrogen signalling, the perception and responses of different plant and microbial cell types to the exchange of ROS signals are likely to be a vital component of inter‐organism relationships. It is essential to understand such processes within the context of the plethora of signalling molecules that contribute to the dialogue between plants and microbes.

## Conflicts of Interest

Christine H. Foyer is the editor‐in‐chief of *Plant, Cell & Environment*, and a co‐author of this article. She was excluded from editorial decision‐making related to the acceptance and publication of this article. Editorial decision‐making was handled independently by editor‐in‐chief Jinxing Lin to minimize bias. The other authors declare no conflicts of interest.

## Data Availability

Data sharing is not applicable to this article as no new data were created or analysed in this study.
